# Validation of the Korean version of the Pubertal Development Scale (PDS-K): a non-invasive self-report tool for epidemiological use

**DOI:** 10.4178/epih.e2025059

**Published:** 2025-10-24

**Authors:** Jeeeun Kim, Dahye Kim, Hyojin Pyun, Woon-Kyeong Jeong, Yuen Mi Cheon, Soo Ji Lee, Joohon Sung

**Affiliations:** 1 Department of Public Health, Graduate School of Public Health, Seoul National University, Seoul, Korea; 2 Institute of Health & Environment, Seoul National University, Seoul, Korea; 3 Department of Family Environment and Welfare, Chonnam National University, College of Human Ecology, Gwangju, Korea

**Keywords:** Puberty, Questionnaires, Adolescent, Epidemiological methods, Validation study

## Abstract

As the average age of pubertal onset continues to decline, the need for reliable and culturally appropriate tools to assess pubertal development has become increasingly important. However, no validated, non-invasive, self-report instrument has been available for use in Korea. This study aimed to translate, culturally adapt, and evaluate the Korean version of the Pubertal Development Scale (PDS-K). The original PDS was translated using a forward–backward translation procedure and reviewed by experts to ensure cultural relevance. The PDS-K was administered to a total of 217 elementary school students (grades 4-6). Internal consistency and test–retest reliability were evaluated using Cronbach’s α, item–total correlations, Cohen’s kappa, and intraclass correlation coefficients (ICC). The PDS-K demonstrated acceptable internal consistency (Cronbach’s α: boys=0.79; girls=0.74) and good test–retest reliability (ICCs: 0.77 for boys; 0.87 for girls). Sex-specific patterns of pubertal progression were also observed. Although further validation across broader age groups and against clinical benchmarks is warranted, the PDS-K provides a practical and culturally adapted tool for the non-invasive assessment of pubertal development and holds promise for large-scale epidemiological research.

## GRAPHICAL ABSTRACT

[Fig f2-epih-47-e2025059]

## Key Message

Despite the rising prevalence of early puberty in Korea, there is currently no culturally appropriate, non-invasive tool for assessing pubertal development in non-clinical settings. This study developed and evaluated the Korean version of the Pubertal Development Scale (PDS-K), demonstrating its reliability and feasibility. The PDS-K also effectively captured sex-specific patterns in pubertal progression. As a brief and self-administered instrument, the PDS-K is a feasible, culturally adapted tool for non-invasive pubertal assessment in Korea, with potential for large-scale use once further validated.

## INTRODUCTION

Puberty is a critical developmental period characterized by rapid biological and psychosocial changes that influence health throughout the life course [1-3]. In Korea, the number of diagnosed cases of precocious puberty increased from 72,152 in 2014 to 186,726 in 2023, corresponding to a 2.6-fold rise according to national insurance data (International Statistical Classification of Diseases and Related Health Problems, 10th revision E301) [4]. While precocious puberty requires clinical diagnosis, the earlier timing of pubertal onset is increasingly observed and carries significant public health implications [5]. These trends highlight the importance of population-based monitoring of pubertal development.

Currently, Korea lacks a standardized, non-invasive screening tool suitable for large-scale epidemiological research. Tanner staging [6], the standard clinical method, requires physical examination and is thus impractical in large, non-clinical settings [7]. Given the variability in pubertal timing and its associations with multiple health outcomes [8,9], there is a pressing need for psychometrically robust, developmentally appropriate, and non-invasive assessment tools for children and adolescents that can be applied in community contexts [10].

The Pubertal Development Scale (PDS) [11,12], a self-report questionnaire, has demonstrated moderate to strong correlations with Tanner staging and has been widely used in international epidemiological research [13-15] because of its simplicity and non-invasive nature. However, no validated Korean version currently exists. As perceptions of pubertal changes can vary across cultural and linguistic contexts, direct translation without adaptation may compromise validity [16]. Therefore, this study aimed to translate and culturally adapt the PDS into Korean (PDS-K) and to evaluate its reliability and validity in Korean adolescents, establishing a feasible screening instrument for epidemiological studies and early detection efforts.

## MATERIALS AND METHODS

### Study design and participants

The initial recruitment included 49 fifth-grade students (ages 10-11). To obtain a more stable and representative dataset, recruitment was expanded to encompass students in grades 4-6 (ages 9-12) from additional schools. According to COnsensus-based Standards for the selection of health status Measurement INstruments guidelines [17], a “very good” sample requires at least 100 participants. In addition, power analyses [18] indicated that minimum sample sizes of approximately 40-80 are sufficient under typical assumptions. The final analytic sample comprised 217 students, with both boys and girls exceeding 100, enabling stratified analyses by sex.

### Measurement instruments

#### PDS

This study employed the PDS-K, adapted from the original PDS. The PDS consists of 5 items with sex-specific questions. Boys responded to items assessing growth spurt, body hair growth, skin changes, voice changes, and facial hair growth. Girls responded to items assessing growth spurt, body hair growth, skin changes, breast development, and menarche status and age at menarche. Each item was rated on a 4-point scale (1=not yet begun, 2=just begun, 3=definitely underway, 4=seems complete), except for menarche, which was coded as 1 (no) or 4 (yes).

The PDS-K included several contextual modifications, such as schematic visual aids for the body and facial hair items and supplementary questions on birth year/month and menarche onset (age and timing). These adaptations were intended to improve comprehension and reporting accuracy while maintaining the original structure and intent of the PDS.

#### PCS

The puberty category score (PCS) classifies individuals into 5 stages (prepubertal, early, mid, late, postpubertal) to improve the interpretability of PDS scores by aligning them with the Tanner staging system [12]. While the original PDS provides a continuous mean score ranging from 1 to 4, this does not directly correspond to the 5 Tanner stages. To address this limitation, the PCS uses a subset of PDS items that specifically reflect secondary sexual characteristics to assign developmental categories. For boys, PCS was based on the summed scores of body hair growth, facial hair growth, and voice changes; for girls, on body hair growth, breast development, and menarche status. In this study, PCS was calculated to describe stage distributions and to enable comparisons with prior studies (detailed scoring criteria are provided in the caption of Table 1).

### Translation and content validity

Following World Health Organization guidelines [19], 2 bilingual experts independently translated the original PDS into Korean. A panel of 5 experts—1 adolescent psychologist, 1 professor of preventive medicine, 2 epidemiologists, and 1 school health nurse—reviewed the draft for semantic equivalence, cultural appropriateness, and clarity. Experts were selected based on their experience in adolescent health research and clinical or public health practice. All items achieved a content validity index of 1.0, following established procedures (Supplementary Material 1).

Additionally, simple schematic drawings were incorporated to enhance comprehension based on feedback from school health and homeroom teachers, who emphasized the need to improve student understanding and reporting accuracy. This adaptation was consistent with prior evidence indicating that visual aids can facilitate comprehension and reduce reporting difficulties in self-administered assessments [20].

For face validity, 6 elementary school students and their parents participated in structured debriefing interviews. Participants confirmed overall comprehensibility and suggested minor clarifications. All adaptations were subsequently finalized under expert panel review, ensuring that the modifications enhanced clarity and usability without altering the original constructs.

### Statistical analysis

Descriptive statistics were used to summarize demographic characteristics and item responses. Internal consistency was assessed using Cronbach’s α and McDonald’s omega (Ωt) to confirm reliability under a 1-factor model. Test–retest reliability was evaluated using the Cohen kappa (item level) and intraclass correlation coefficients (ICC, total score) based on a 2-way mixed-effects model. Reliability thresholds were applied as follows: Cronbach’s α≥0.70 as acceptable [21]; Cohen’s kappa <0.20=slight, 0.21-0.40=fair, 0.41-0.60=moderate, 0.61-0.80=substantial, >0.80=almost perfect [22]; ICC <0.50=poor, 0.50-0.75=moderate, 0.75-0.90=good, >0.90=excellent [23]. Height and body mass index (BMI) were examined descriptively across sex and pubertal categories to contextualize developmental status. Additionally, the association between PDS-K scores and PCS stages was tested using analysis of variance (ANOVA) with a linear trend, with detailed results reported in the Supplement. All analyses were conducted using R version 4.3.0 (R Foundation for Statistical Computing, Vienna, Austria), with p-value <0.05 considered statistically significant.

### Ethics statement

The study protocol was approved by the Institutional Review Board of Seoul National University (IRB No. 1311-045-533).

## RESULTS

### Participant characteristics

A total of 217 students were included in the analysis (105 boys and 112 girls). The mean age was slightly higher among girls than boys (11.51 vs. 11.14 years, p=0.019). Among anthropometric measures, only BMI differed significantly, being higher in boys than girls (19.61 vs. 18.37 kg/m², p=0.008). PDS-K scores were also significantly higher in girls than boys (1.8 vs. 1.4, p<0.001). Based on PCS stages, most boys were classified as prepubertal (73.3%), whereas girls were more evenly distributed across stages, with nearly one-fifth classified as being in late puberty (p<0.001) (Table 1, Supplementary Material 2). PDS-K scores increased monotonically across PCS stages (ANOVA p<0.001), indicating a consistent positive association (Supplementary Materials 3 and 4).

### Reliability of the Korean version of the Pubertal Development Scale

Table 2 summarizes the reliability results of the PDS-K. Internal consistency exceeded the conventional threshold of 0.70 in both sexes (Cronbach’s α=0.79 for boys; 0.74 for girls). Ωt was 0.81 for boys and 0.75 for girls. ICCs demonstrated substantial agreement in boys (0.77; 95% confidence interval, 0.60 to 0.86) and almost perfect agreement in girls (0.87; 95% CI, 0.78 to 0.92). Most items showed moderate to strong item–total correlations (>0.50), except for growth spurt (boys: 0.36; girls: 0.44) and breast development in girls (0.48). Test–retest reliability over 1 month ranged from moderate to excellent (κ=0.51-0.85 in boys; 0.58-0.90 in girls).

### Association between Korean version of the Pubertal Development Scale scores and growth indicators

**Figure 1.**
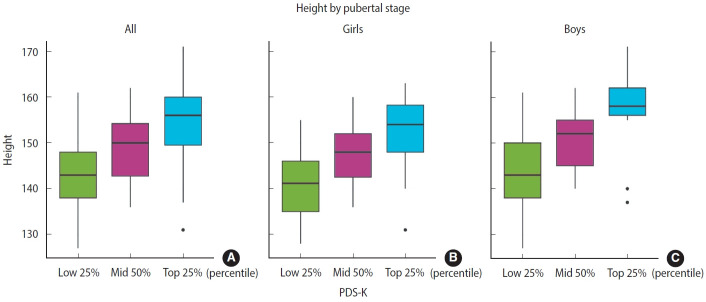
Height distribution across Korean version of the Pubertal Development Scale (PDS-K) score percentiles by sex. Box plots illustrate height across PDS-K score percentiles (lowest 25%, middle 50%, highest 25%) for the total sample (A), girls (B), and boys (C). Boxes indicate the interquartile range, with median lines shown; outliers are plotted as individual points.

Figure 1 and Table 3 present growth indicators across PDS-K scores. Height increased significantly with higher PDS-K scores in both sexes. BMI exhibited sex-specific patterns: it did not vary significantly among boys (p=0.198) but increased steadily among girls (p<0.001). Supplementary analyses using PCS stages yielded consistent findings: height increased with advancing PCS stage in both sexes, whereas BMI rose significantly across stages only among girls (Supplementary Materials 5-8).

## DISCUSSION

This study evaluated the reliability and validity of the PDS-K and its applicability as a self-reported screening tool among Korean adolescents. Reliability indices, including Cronbach’s α and ICC, indicated acceptable internal consistency and temporal stability, supporting its use in epidemiological contexts.

A key contribution of this study is the demonstrated utility of the PDS-K in large-scale, non-clinical settings. Its self-report format reduces the ethical and logistical challenges of physical examination while effectively capturing meaningful developmental patterns. Prior research has highlighted the feasibility of self-reported pubertal assessment [24], and the present findings extend that evidence to a validated Korean version.

Compared with self-reported Tanner staging, which requires sensitive visual materials and typically shows only moderate agreement with clinician ratings [25], the PDS-K incorporates both general (e.g., growth spurt, skin changes) and sex-specific indicators in non-sensitive formats. These were supplemented with simple schematic drawings to facilitate comprehension. Such adaptations enhance cultural appropriateness, reduce discomfort, and minimize missing data.

Moreover, the PDS is highly adaptable to different informants and research purposes. It can be used as a continuous score, categorized into developmental stages through the PCS, or focused on specific items such as age at menarche [26,27]. In this study, the PCS was applied to describe stage distributions and to compare sex-specific patterns, ensuring comparability with prior research. The PDS has also been used in large-scale international initiatives such as the Adolescent Brain Cognitive Development (ABCD) Study, where caregiver reports supplement self-reports at younger ages to address the limitations of children’s self-assessments [28]. These examples underscore its versatility and broad acceptance as a non-invasive tool for population-based research.

At the item level, some variability was noted. The growth-spurt item demonstrated weaker item–total correlations and lower retest agreement, consistent with earlier reports that gradual somatic changes are difficult for adolescents to recognize [10]. In contrast, menarche and facial hair growth items showed strong reliability, whereas breast development exhibited only moderate associations with total scores. These findings may reflect both measurement challenges and genuine individual differences in the timing of pubertal changes. Thus, although the scale-level indices confirm that the PDS-K is coherent and reproducible, item-level results highlight potential areas for refinement and suggest its usefulness for exploring variability across specific developmental domains.

Beyond psychometric properties, the PDS-K also reflected expected developmental trends: height increased with higher PDS-K scores in both sexes, while BMI rose significantly in girls but remained stable in boys. Although these indices are not clinical benchmarks, they align with established growth trajectories [1] and provide valuable context for interpreting developmental patterns in population studies.

This study represents the first independent validation of the PDS-K. Its simplicity, non-invasiveness, and developmental appropriateness support its use in large-scale surveys. Nonetheless, several limitations warrant consideration. The sample consisted of students in grades 4-6, capturing the onset of pubertal changes but limiting generalizability to older adolescents. Although the sample size was adequate for stable estimates, larger and more diverse cohorts would enhance generalizability and allow finer examination of sex- and stage-specific response patterns.

Additionally, the PDS itself has recognized limitations. It does not directly correspond to Tanner staging, is less sensitive to early pubertal changes, and often demonstrates systematic discrepancies relative to clinician ratings—wherein less advanced adolescents tend to overestimate and more advanced adolescents tend to underestimate their stage. These issues underscore the need for cautious interpretation when using self-reported measures alone.

Finally, this study did not include clinical or hormonal benchmarks. Given prior findings of lower agreement between PDS and Tanner staging compared with clinician assessments [27], these results should be interpreted conservatively. Future research incorporating biological markers and longitudinal designs will be essential to establish long-term validity and clinical relevance. Moreover, while the PCS provided a practical categorical framework for describing stage distributions, it is derived directly from PDS items and lacks independent validation in Korean adolescents. Its use should therefore be considered exploratory rather than confirmatory. In line with prior recommendations [27], we suggest applying this approach primarily to capture general developmental trajectories rather than approximate clinical staging.

Taken together, these findings indicate that the PDS-K is a feasible and reliable tool for non-invasive assessment of pubertal status in Korean adolescents. It offers a practical, culturally relevant alternative to clinician-based methods for epidemiological research, though its use should be viewed as complementary rather than definitive given current limitations.

## Figures and Tables

**Figure f2-epih-47-e2025059:**
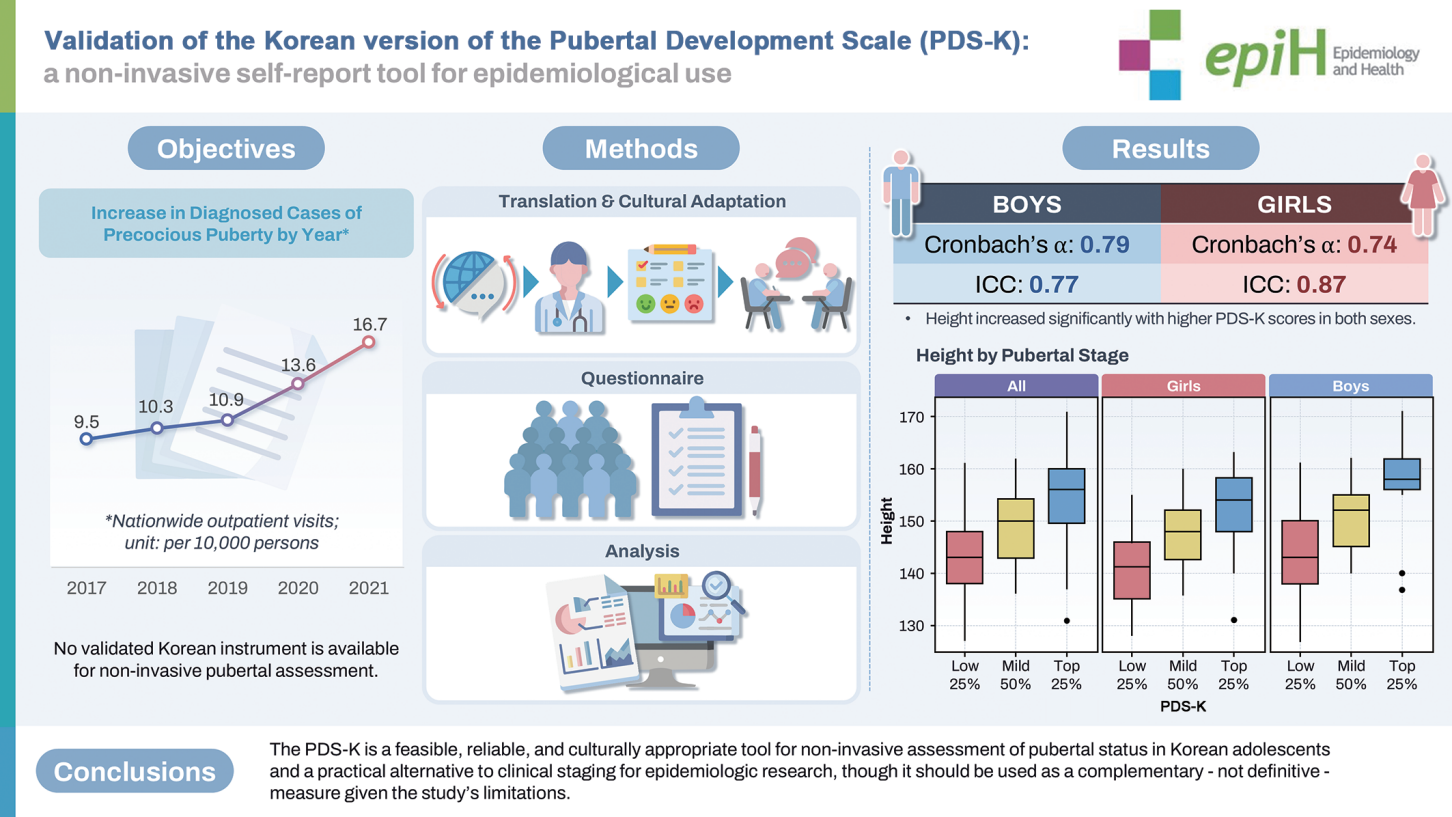


**Table 1.** Demographic and pubertal characteristics of participants by sex^1^

Characteristics	Boys (n=105)	Girls (n=112)	p-value^2^
Age (yr)	11.14±0.87	11.51±1.38	0.019
Height (cm)	146.88±9.32	147.92±8.21	0.385
Weight (kg)	42.90±11.42	40.77±9.28	0.136
Body mass index (kg/m²)	19.61±3.65	18.37±3.09	0.008
Pubertal characteristics			
Growth spurt (underway/finished)	53 (51.0)	66 (58.9)	0.274
Skin change (underway/finished)	10 (9.6)	15 (13.4)	0.404
Body hair growth (underway/finished)	1 (1.0)	6 (5.4)	0.121
Specific pubertal characteristics			
Male			
Facial hair growth (underway/finished)	4 (3.9)	-	
Voice change (underway/finished)	6 (5.8)	-	
Female			
Breast development (underway/finished)	-	40 (35.7)	
Menarche (yes)	-	22 (19.6)	
Age at menarche	-	10.91±0.68	
PDS-K scores	1.4±0.4	1.8±0.5	<0.001
Puberty category score			<0.001
Prepubertal	77 (73.3)	22 (19.6)	
Early puberty	22 (21.6)	36 (32.1)	
Midpubertal	6 (5.9)	32 (28.6)	
Late puberty	0 (0)	22 (19.6)	

Values are presented as mean±standard deviation or number (%). PDS-K, Korean version of the Pubertal Development Scale.

1 “Underway/finished” indicates participants who reported that a pubertal characteristic was currently occurring or completed; Facial hair growth and voice change were assessed only in boys; breast development and menarche only in girls; Age at menarche is reported only for those who had experienced menarche; Boys: Prepubertal=3; Early=4-5; Mid=6-8; Late=9-11; Post=12. Girls: Prepubertal=2 (no menarche); Early=3 (no menarche); Mid=5 (no menarche); Late=≤7 (with menarche); Post=8 (with menarche).

2 Using independent t-tests (continuous) and chi-square tests (categorical).

**Table 2.** Item analysis, internal consistency, and test-retest reliability of the Korean version of the Pubertal Development Scale (PDS-K)^1^

Items	Scores	Item-total correlation	Cronbach’s α (95% CI)	Cronbach’s α if item deleted	1-mo test-retest reliability, Cohen’s kappa (95% CI)
Boys					
Growth spurt	2.15±0.96	0.36	-	0.83	0.51
Body hair growth	1.12±0.36	0.72	-	0.73	0.85
Skin change	1.38±0.67	0.77	-	0.72	0.75
Voice change	1.18±0.54	0.58	-	0.78	0.74
Facial hair growth	1.20±0.53	0.85	-	0.69	0.80
PDS-K scores	1.42±0.36	-	0.79 (0.73, 0.86)	-	ICC (2,1)=0.77 (0.60, 0.86)
Girls					
Growth spurt	2.33±1.03	0.44	-	0.76	0.58
Body hair growth	1.28±0.62	0.57	-	0.69	0.78
Skin change	1.62±0.76	0.65	-	0.67	0.81
Breast development	2.14±0.75	0.48	-	0.72	0.90
Menarche	1.59±1.20	0.88	-	0.58	0.88
PDS-K scores	1.81±0.54	-	0.74 (0.58, 0.79)	-	ICC (2,1)=0.87 (0.78, 0.92)

Values are presented as mean±standard deviation. CI, confidence interval; ICC, intraclass correlation coefficient.

1 Item–total correlations represent the association of each item with the overall scale, based on the correlation between the item and the total score excluding that item; Cronbach’s α reflects internal consistency; “α if item deleted” shows reliability if the item is removed; Test-retest reliability was assessed using Cohen’s κ (items) and the ICC (total score). ICC interpretation [23]: poor <0.50, moderate 0.50-0.75, good 0.75-0.90, excellent >0.90.

**Table 3.** Associations between PDS-K scores and growth indicators by sex^1^

Sex	Outcome variable	β (95% CI)^2^	p-value
Boys	Height (cm)	15.13 (10.98, 19.29)	<0.001
	BMI (kg/m²)	1.30 (-0.69, 3.29)	0.198
Girls	Height (cm)	8.62 (6.27, 10.98)	<0.001
	BMI (kg/m²)	1.98 (0.96, 3.00)	<0.001

PDS-K, Korean version of the Pubertal Development Scale; CI, confidence interval; BMI, body mass index.

1 Linear regression analysis was performed using PDS-K scores as a continuous predictor for height and BMI, stratified by sex.

2 β indicates unstandardized regression coefficient.

## Supplementary materials

Supplementary Material 1.

Supplementary Material 2. Distribution of PDS-K item responses by sex and time point (first and second survey)

Supplementary Material 3. Association between PDS-K scores and PCS

Supplementary Material 4. Association between PDS-K scores and PCS.

Supplementary Material 5. Associations between pubertal stage (PCS) and growth indicators (height and BMI) by sex

Supplementary Material 6. Associations between pubertal development score (PDS-K) and growth indicators in the overall population.

Supplementary Material 7. Associations between pubertal development score (PDS-K) and growth indicators by sex.

Supplementary Material 8. Distribution of height by pubertal stage (overall and by sex)
