# Supplementary material for: Validation of the Korean version of the Pubertal Development Scale (PDS-K): a non-invasive self-report tool for epidemiological use
Source: Epidemiol Health. 2025 Oct 24;47:e2025059. doi: 10.4178/epih.e2025059 (PMC12869118; doi:10.4178/epih.e2025059)
Supplement: Supplementary Material 1. [file epih-47-e2025059-Supplementary-1.docx]

**Supplementary Material 1**

Lynn MR. Determination and quantification of content validity. Nurs Res. 1986 Nov-Dec;35(6):382-5.

Polit DF, Beck CT, Owen SV. Is the CVI an acceptable indicator of content validity? Appraisal and recommendations. Res Nurs Health. 2007 Aug;30(4):459-67. doi: 10.1002/nur.20199.
