# Supplementary material for: Validation of the Korean version of the Pubertal Development Scale (PDS-K): a non-invasive self-report tool for epidemiological use
Source: Epidemiol Health. 2025 Oct 24;47:e2025059. doi: 10.4178/epih.e2025059 (PMC12869118; doi:10.4178/epih.e2025059)
Supplement: Supplementary Material 2. — Distribution of PDS-K item responses by sex and time point (first and second survey) [file epih-47-e2025059-Supplementary-2.docx]

**Supplementary Material 2.**

**Distribution of PDS-K item responses by sex and time point (first and second survey)**

|  | Boys (N=105) | | Girls (N=112) | |
| --- | --- | --- | --- | --- |
|  | 1st | 2nd | 1st | 2nd |
| ***Growth spurt*** |  |  |  |  |
| Don’t know | 4 (4%) | 7 (7%) | 5 (4%) | 1 (1%) |
| Not begun | 29 (28%) | 12(14%) | 25 (22%) | 18 (19%) |
| Barely begun | 19(17%) | 17 (20%) | 16 (14%) | 23 (24%) |
| Underway | 53 (51%) | 52 (60%) | 60 (54%) | 48 (51%) |
| Has finished | 0 (0%) | 1 (1%) | 6 (5%) | 5 (5%) |
| ***Body hair growth*** |  |  |  |  |
| Don’t know | 0 (0%) | 0 (0%) | 0 (0%) | 0 (0%) |
| Not begun | 92 (88%) | 74 (86%) | 89 (79%) | 65 (68%) |
| Barely begun | 11 (11%) | 11 (13%) | 17 (15%) | 20 (21%) |
| Underway | 1 (1%) | 1 (1%) | 4 (4%) | 9 (9%) |
| Has finished | 0 (0%) | 0 (0%) | 2 (2%) | 1 (1%) |
| ***Skin change*** |  |  |  |  |
| Don’t know | 1 (1%) | 1 (1%) | 0 (0%) | 0 (0%) |
| Not begun | 74 (70%) | 50 (57%) | 59 (53%) | 37 (39%) |
| Barely begun | 20 (19%) | 30 (34%) | 38 (34%) | 44 (46%) |
| Underway | 10 (10%) | 5 (6%) | 13 (12%) | 12 (13%) |
| Has finished | 0 (0%) | 1 (1%) | 2 (2%) | 2 (2%) |
| ***Voice change/Breast development*** | | | | |
| Don’t know | 1 (1%) | 3 (3%) | 0 (0%) | 0 (0%) |
| Not begun | 90(86%) | 64 (74%) | 24 (21%) | 17 (18%) |
| Barely begun | 8 (8%) | 13 (15%) | 48 (43%) | 38 (40%) |
| Underway | 6 (6%) | 7 (8%) | 40 (36%) | 40 (42%) |
| Has finished | 0 (0%) | 0 (0%) | 0 (0%) | 1 (1%) |
| ***Facial hair growth/ Menarche*** | | | | |
| Don’t know | 1 (1%) | 5 (6%) |  |  |
| Not begun /No | 87 (84%) | 68 (79%) | 90 (80%) | 73 (76%) |
| Barely begun | 12 (12%) | 11 (13%) |  |  |
| Underway | 3 (3%) | 5 (6%) |  |  |
| Has finished /Yes | 1 (1%) | 0 (0%) | 22 (20%) | 23 (24%) |

**Note:** Values are presented as mean ± standard deviation or number (%)."1st" and "2nd" refer to responses collected at the first and second time points, respectively. For each item, response options were: *Don’t know*, *Not begun*, *Barely begun*, *Underway*, and *Has finished*. For the menarche item (girls), response options were *Yes* or *No*.
