# Supplementary material for: Validation of the Korean version of the Pubertal Development Scale (PDS-K): a non-invasive self-report tool for epidemiological use
Source: Epidemiol Health. 2025 Oct 24;47:e2025059. doi: 10.4178/epih.e2025059 (PMC12869118; doi:10.4178/epih.e2025059)
Supplement: Supplementary Material 3. — Association between PDS-K scores and PCS [file epih-47-e2025059-Supplementary-3.docx]

**Supplementary Material 3**

| (a)  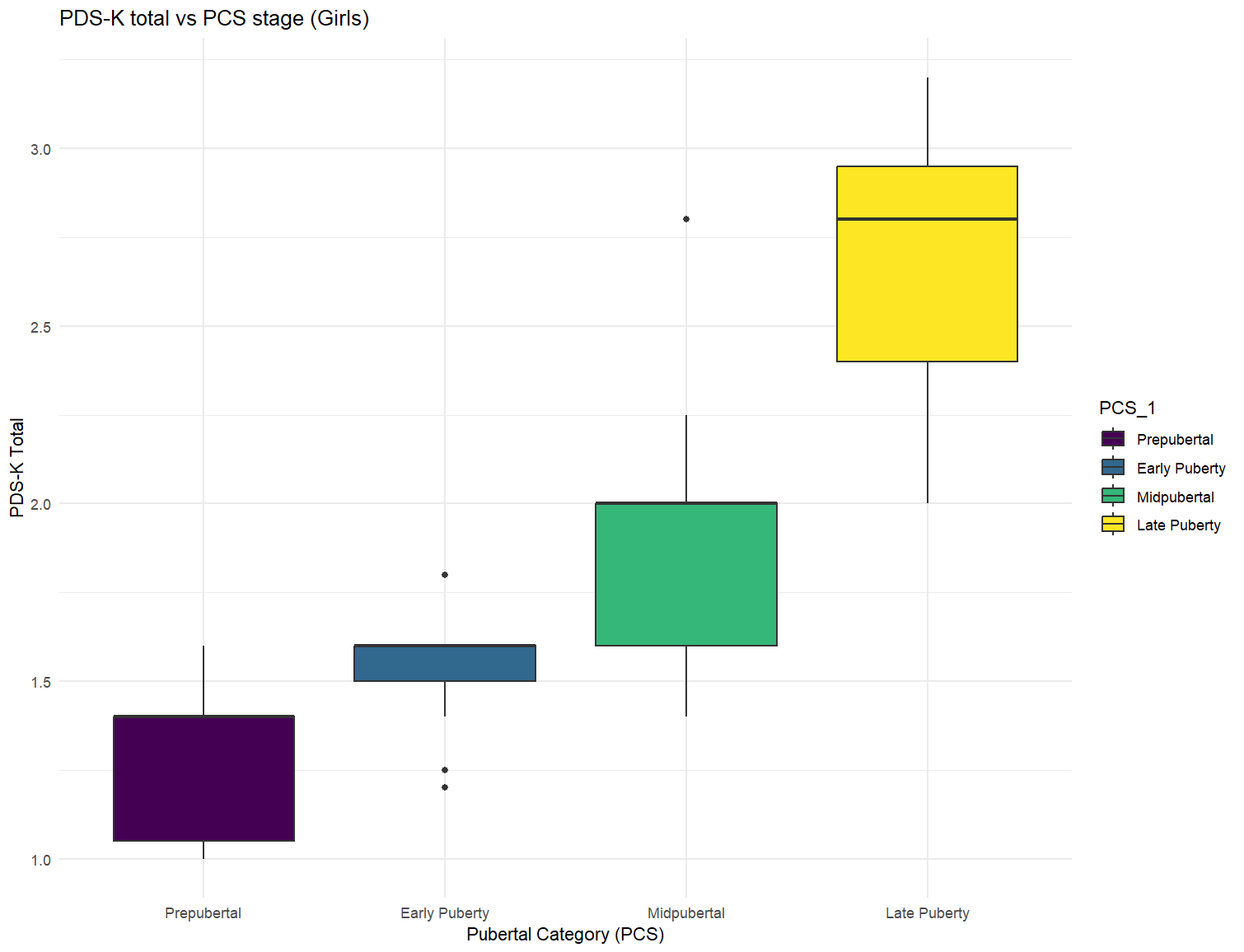 |
| --- |
| (b)  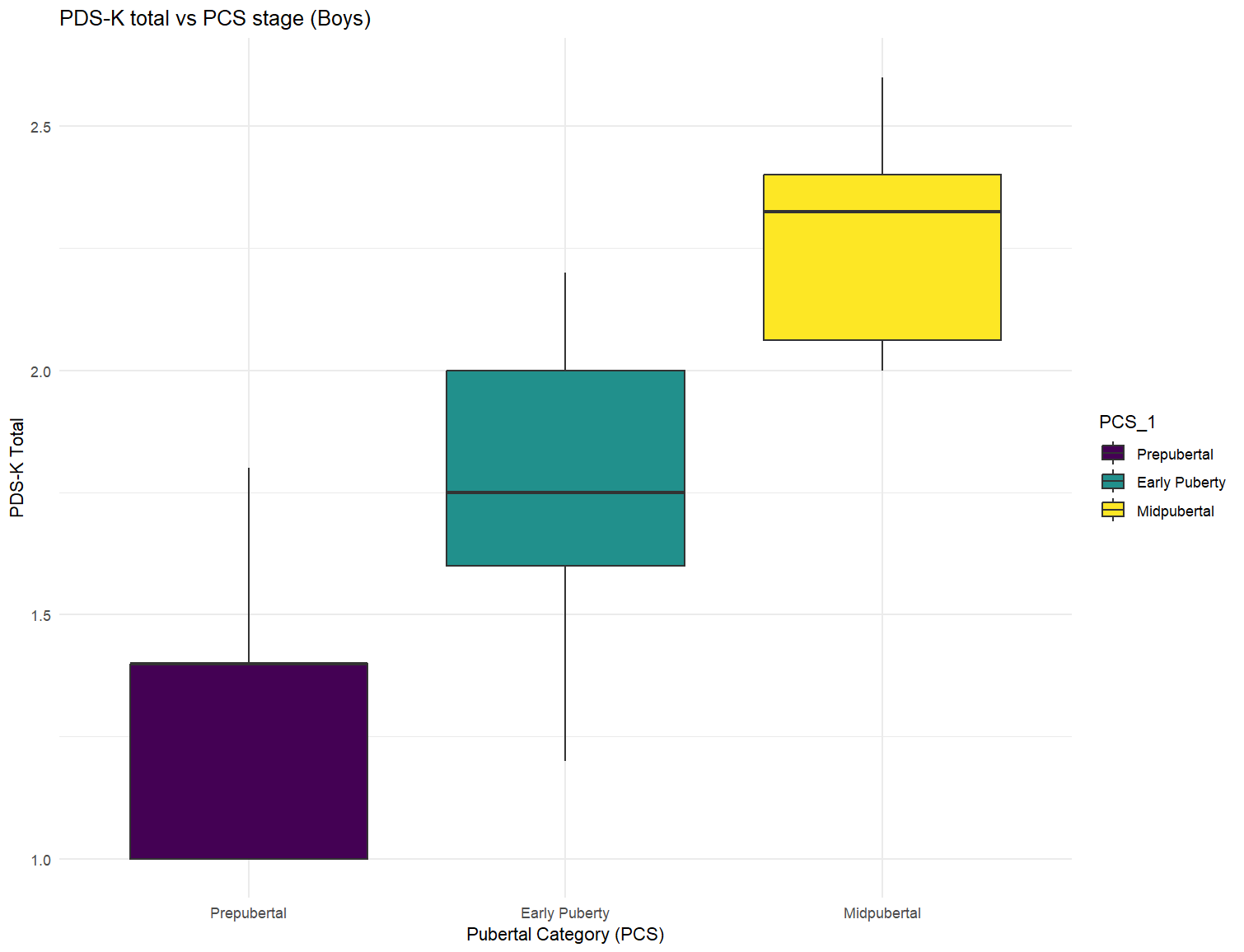 |

**Association between PDS-K scores and PCS.** Boxplots show the distribution of PDS-K scores across pubertal stages (PCS) separately for boys (left) and girls (right). PDS-K scores increased monotonically with advancing PCS, indicating a consistent positive association (ANOVA, p < 0.001).
