# Supplementary material for: Validation of the Korean version of the Pubertal Development Scale (PDS-K): a non-invasive self-report tool for epidemiological use
Source: Epidemiol Health. 2025 Oct 24;47:e2025059. doi: 10.4178/epih.e2025059 (PMC12869118; doi:10.4178/epih.e2025059)
Supplement: Supplementary Material 4. — Association between PDS-K scores and PCS. [file epih-47-e2025059-Supplementary-4.docx]

**Supplementary Material 4**

**Association between PDS-K scores and PCS.**

| **Sex** | **PCS** | **N** | **PDS-K** | **ANOVA p-value** | **Kruskal–Wallis**  **p-value** | **Linear trend**  **(β, SE)** |
| --- | --- | --- | --- | --- | --- | --- |
| Boys | Prepubertal | 77 | 1.27±0.20 | <0.001 | <0.001 | β = 0.48 (0.04), p<0.001 |
|  | Early Puberty | 22 | 1.72±0.30 |  |  |  |
|  | Midpubertal | 6 | 2.28±0.24 |  |  |  |
| Girls | Prepubertal | 22 | 1.31±0.22 | <0.001 | <0.001 | β = 0.44 (0.03), p<0.001 |
|  | Early Puberty | 36 | 1.53±0.18 |  |  |  |
|  | Midpubertal | 32 | 1.88±0.29 |  |  |  |
|  | Late Puberty | 22 | 2.68±0.35 |  |  |  |

Note: ANOVA, Kruskal–Wallis, and linear trend tests assess overall differences across PCS stages within each sex. Values are presented as Mean ± SD. Regression coefficients (β, 95% CI) were derived from linear regression models, using the prepubertal stage as reference
