# Supplementary material for: Validation of the Korean version of the Pubertal Development Scale (PDS-K): a non-invasive self-report tool for epidemiological use
Source: Epidemiol Health. 2025 Oct 24;47:e2025059. doi: 10.4178/epih.e2025059 (PMC12869118; doi:10.4178/epih.e2025059)
Supplement: Supplementary Material 5. — Associations between pubertal stage (PCS) and growth indicators (height and BMI) by sex [file epih-47-e2025059-Supplementary-5.docx]

**Supplementary Material 5**

**Associations between pubertal stage (PCS) and growth indicators (height and BMI) by sex**

|  | Height  (Mean; SD, cm) | Height Regression  (β, 95% CI, p) | BMI  (Mean; SD, kg/m²) | BMI Regression  (β, 95% CI, p) |
| --- | --- | --- | --- | --- |
| *Boys* |  |  |  |  |
| Prepubertal | 144.31 ± 8.13 | Reference | 19.34 ± 3.61 | Reference |
| Early Puberty | 152.94 ± 7.70 | **8.63 (4.09, 13.16,**  **p = <0.001)** | 20.45 ± 3.79 | 1.11 (-0.89, 3.11,  p = 0.359) |
| Midpubertal | 158.33 ± 11.96 | **14.02 (6.10, 21.95,**  **p = <0.001)** | 19.79 ± 3.62 | 0.45 (-3.04, 3.94,  p = 0.928) |
| p for trend |  | **<0.001** |  | 0.335 |
| *Girls* |  |  |  |  |
| Prepubertal | 141.37 ± 8.35 | Reference | 16.79 ± 2.74 | Reference |
| Early Puberty | 146.44 ± 6.98 | **5.07 (0.58, 9.56, p = 0.022)** | 17.81 ± 2.83 | 1.03 (-0.90, 2.96,  p = 0.441) |
| Midpubertal | 148.94 ± 6.40 | **7.57 (2.98, 12.16,**  **p = <0.001)** | 18.94 ± 3.31 | **2.16 (0.18, 4.13,**  **p = 0.028)** |
| Late Puberty | 155.41 ± 5.95 | **14.04 (9.04, 19.04,**  **p = <0.001)** | 19.95 ± 2.69 | **3.16 (1.02, 5.31,**  **p = 0.002)** |
| p for trend |  | **<0.001** |  | **<0.001** |

Note: Values are presented as Mean ± SD. Regression coefficients (β, 95% CI) were derived from linear regression models, using the prepubertal stage as reference. P for trend was calculated treating stage as ordinal. Significant results (p < 0.05) are in bold.
