# Supplementary material for: Validation of the Korean version of the Pubertal Development Scale (PDS-K): a non-invasive self-report tool for epidemiological use
Source: Epidemiol Health. 2025 Oct 24;47:e2025059. doi: 10.4178/epih.e2025059 (PMC12869118; doi:10.4178/epih.e2025059)
Supplement: Supplementary Material 6. — Associations between pubertal development score (PDS-K) and growth indicators in the overall population. [file epih-47-e2025059-Supplementary-6.docx]

**Supplementary Material 6**


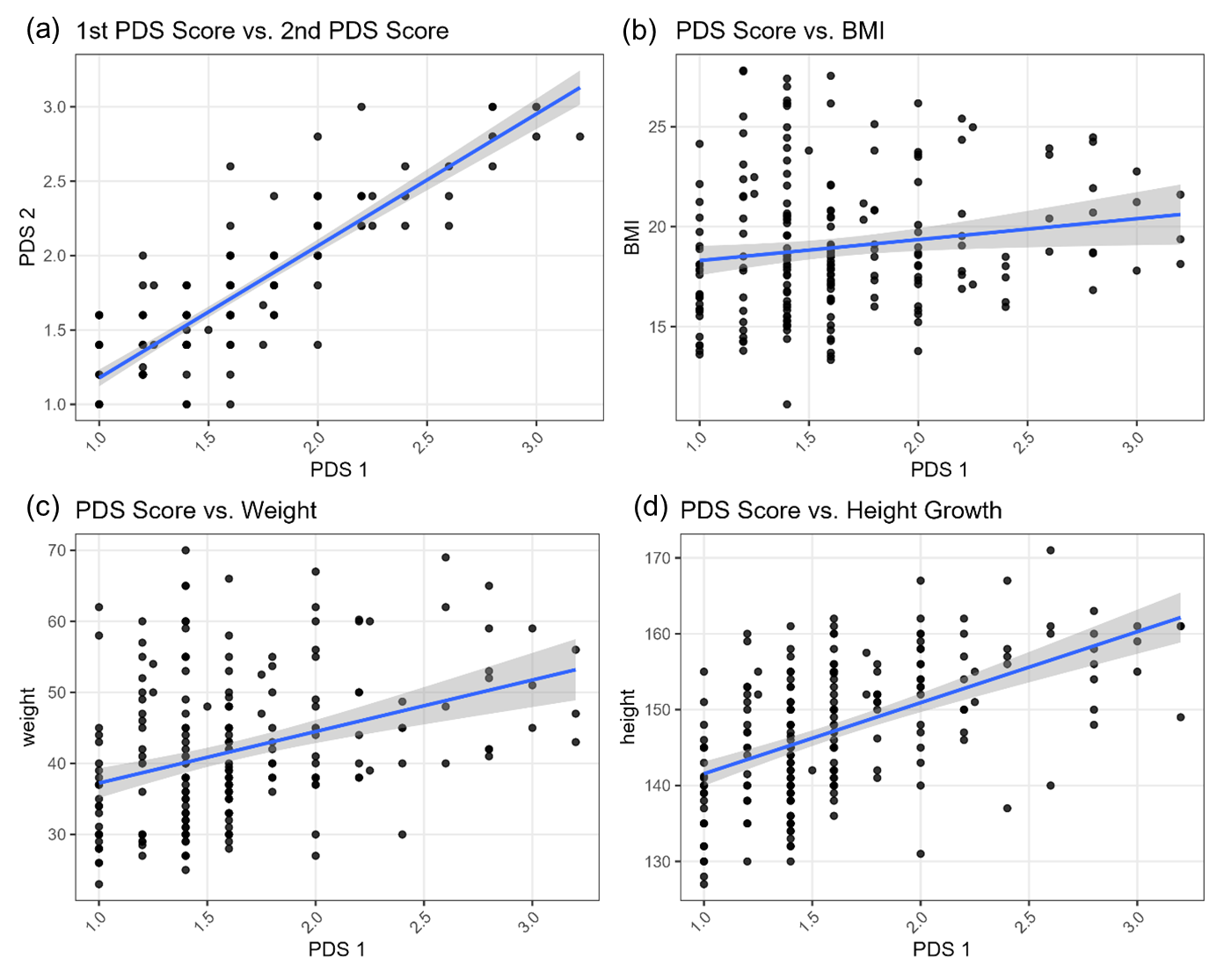


**Associations between pubertal development score (PDS-K) and growth indicators in the overall population.** Scatter plots with regression lines and 95% confidence intervals (shaded area) illustrating the associations between PDS-K and growth indicators in the overall participants. (a) correlation between the first and second PDS-K, (b) PDS-K vs. BMI., (c) PDS-K vs. Weight, (d) PDS-K vs. Height Growth
