# Supplementary material for: Validation of the Korean version of the Pubertal Development Scale (PDS-K): a non-invasive self-report tool for epidemiological use
Source: Epidemiol Health. 2025 Oct 24;47:e2025059. doi: 10.4178/epih.e2025059 (PMC12869118; doi:10.4178/epih.e2025059)
Supplement: Supplementary Material 7. — Associations between pubertal development score (PDS-K) and growth indicators by sex. [file epih-47-e2025059-Supplementary-7.docx]

**Supplementary Material 7**

**
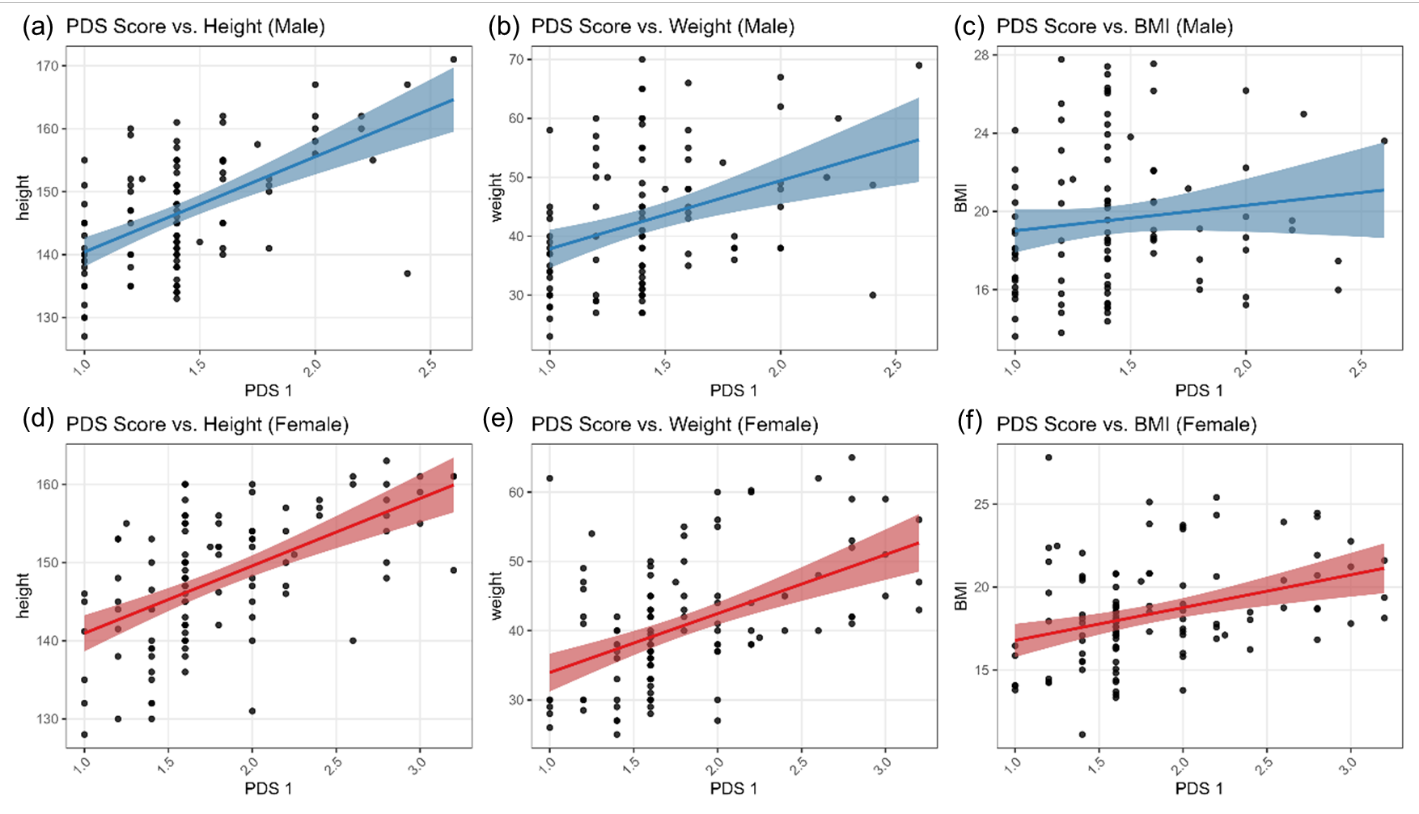
**

**Associations between pubertal development score (PDS-K) and growth indicators by sex.** Scatter plots with regression lines and 95% confidence intervals (shaded area) illustrating the associations between PDS-K and growth indicators (Height, Weight, and BMI) stratified by sex. (a) PDS-K vs. Height in Males, (b) PDS-K vs. Weight in Males (c) PDS-K vs. BMI in Males, (d) PDS-K vs. Height in Females, (e) PDS-K vs. Weight in Females, (f) PDS-K vs. BMI in Females.
