# Supplementary material for: Validation of the Korean version of the Pubertal Development Scale (PDS-K): a non-invasive self-report tool for epidemiological use
Source: Epidemiol Health. 2025 Oct 24;47:e2025059. doi: 10.4178/epih.e2025059 (PMC12869118; doi:10.4178/epih.e2025059)
Supplement: Supplementary Material 8. — Distribution of height by pubertal stage (overall and by sex) [file epih-47-e2025059-Supplementary-8.docx]

**Supplementary Material 8**


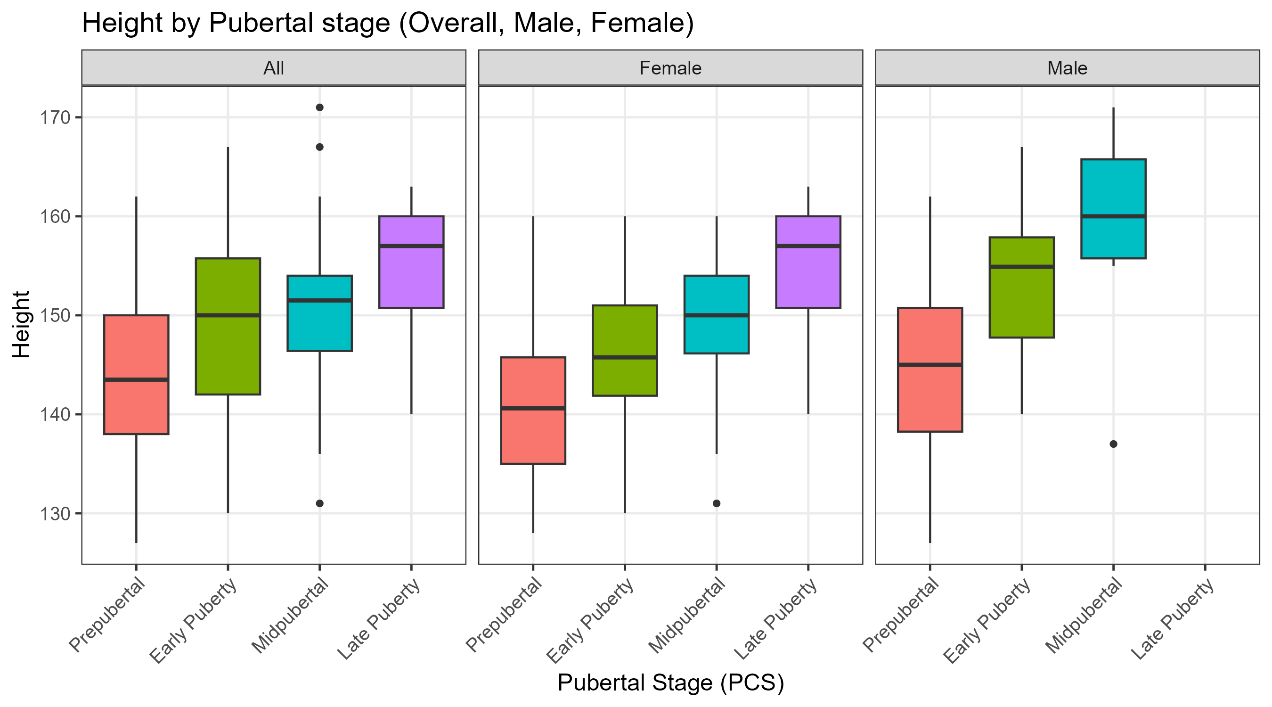


**Distribution of height by pubertal stage (overall and by sex).** Box plots illustrating the distribution of height across pubertal stages (PCS) for the total sample (left), females (middle), and males (right). Median height and interquartile ranges are displayed, with outliers represented as individual points.
